# Preventive brain radio-chemotherapy alters plasticity associated metabolite profile in the hippocampus but seems to not affect spatial memory in young leukemia patients

**DOI:** 10.1002/brb3.368

**Published:** 2015-07-14

**Authors:** Moritz D Brandt, Kalina Brandt, Annett Werner, Robby Schönfeld, Kai Loewenbrück, Markus Donix, Markus Schaich, Martin Bornhäuser, Rüdiger von Kummer, Bernd Leplow, Alexander Storch

**Affiliations:** 1 Division of Neurodegenerative Diseases, Department of Neurology, Technische Universität Dresden01307, Dresden, Germany; 2 German Center for Neurodegenerative Diseases (DZNE) Dresden10307, Dresden, Germany; 3 Center for Regenerative Therapies Dresden (CRTD), Technische Universität Dresden01307, Dresden, Germany; 4 Department of Haematology and Oncology, University Hospital Dresden01307, Dresden, Germany; 5 Department of Neuroradiology, Technische Universität Dresden01307, Dresden, Germany; 6 Institute for Psychology, University of HalleHalle, Germany; 7 Department of Psychiatry, University Hospital Dresden01307, Dresden, Germany

**Keywords:** Adult neurogenesis, choline, human, proton magnetic resonance spectroscopy, virtual water maze

## Abstract

**Background:**

Neuronal plasticity leading to evolving reorganization of the neuronal network during entire lifespan plays an important role for brain function especially memory performance. Adult neurogenesis occurring in the dentate gyrus of the hippocampus represents the maximal way of network reorganization. Brain radio-chemotherapy strongly inhibits adult hippocampal neurogenesis in mice leading to impaired spatial memory.

**Methods:**

To elucidate the effects of CNS radio-chemotherapy on hippocampal plasticity and function in humans, we performed a longitudinal pilot study using 3T proton magnetic resonance spectroscopy (^1^H-MRS) and virtual water-maze-tests in 10 de-novo patients with acute lymphoblastic leukemia undergoing preventive whole brain radio-chemotherapy. Patients were examined before, during and after treatment.

**Results:**

CNS radio-chemotherapy did neither affect recall performance in probe trails nor flexible (reversal) relearning of a new target position over a time frame of 10 weeks measured by longitudinal virtual water-maze-testing, but provoked hippocampus-specific decrease in choline as a metabolite associated with cellular plasticity in ^1^H-MRS.

**Conclusion:**

Albeit this pilot study needs to be followed up to definitely resolve the question about the functional role of adult human neurogenesis, the presented data suggest that ^1^H-MRS allows the detection of neurogenesis-associated plasticity in the human brain.

## Introduction

Cellular plasticity in the brain is a major prerequisite at structural level for acquisition of new memory. Within the hippocampus we have the special situation that plasticity is not only restricted to synapses but also to the generation of new neurons that are integrated into the preexisting network. The functional relevance of these new neurons as well as the putative role of adult neurogenesis in diseases that are accompanied by hippocampal dysfunction has been investigated intensively in animal models (Kempermann and Kronenberg 2003; Jessberger et al. 2005; Garthe et al. 2009; Deng et al. 2010). Adult neurogenesis is suggested to be essential for a specific aspect of memory formation called pattern separation. Mice that lack adult neurogenesis and were trained in the Morris Water Maze task (a spatial memory task) have difficulties in reversal learning a new target position indicating that new neurons are important to integrate novel information into existing networks (Garthe et al. 2009). Moreover several animal studies revealed a functional link between adult hippocampal neurogenesis and depression, epilepsy and dementia (for overview see: Braun and Jessberger 2014). Although there is substantial adult hippocampal neurogenesis in the human brain (Eriksson et al. 1998; Spalding et al. 2013), to date no reliable method exists to visualize or even quantify adult human neurogenesis in vivo. Thus the functional relevance of human neurogenesis as well as its role in diseases accompanied by hippocampal dysfunction are still unknown. Animal studies that investigated the functional role of adult neurogenesis often use hippocampus radiation or CNS-chemotherapy to inhibit adult stem- and precursor cell proliferation (Monje 2008; Burghardt et al. 2012). Whether those treatments cause hippocampal dysfunctions in cancer patients due to treatment induced reduction in neurogenesis has been speculated, but could not have been proven.

These open questions taken together with the opportunity to examine the effects of radio-chemotherapy on healthy young human brain in patients suffering from newly diagnosed acute lymphoblastic leukemia (ALL) routinely allocated to preventive brain radio-chemotherapy prompted our investigation on how indicators of adult hippocampal neurogenesis in humans is affected by brain radiation and intrathecal chemotherapy known to block adult neurogenesis in mice. We used the choline peak in proton magnetic resonance spectroscopy (^1^H-MRS) within the hippocampal tissue as a measure of altered cell membrane turnover associated with proliferating cells, synaptogenesis and axon sprouting (Araki and Wurtman 1997; Sartorius et al. 2003) and a Virtual Morris Water Maze (VWM) to determine spatial memory performance before, during and after radio-chemotherapy as indicators for neurogenesis-associated changes.

## Subjects and Methods

**Figure 1:**
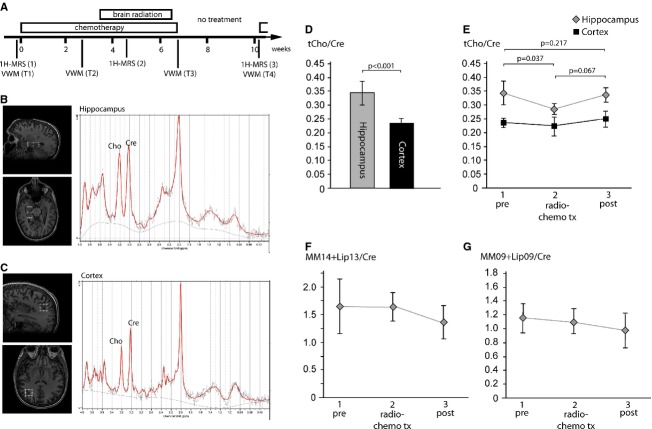
^1^H-MRS in human brain radio-chemotherapy. (A) Time line of the study protocol depicting the Virtual Water Maze (VWM) tests and ^1^H-MRS measurements with respect to the radio-chemotherapy treatment period. (B, C) Examples of a ^1^H-MRS spectrum edited with LCModel software of the hippocampus (B) and cortex (C). (D) The tCho/Cre ratio as a cell proliferation marker is higher in the hippocampus compared to cortex before radio-chemotherapy. (E) The tCho/Cre ratio selectively declines in the hippocampus (*F*-value = 3.930; *P *= 0.041; one-way repeated-measure ANOVA of the intent-to-treat population), but not in the cortex during radio-chemotherapy (*F*-value = 0.400; *P *= 0.677). *P*-values in diagram represent the result from post-hoc Bonferroni-adjusted two-sided paired *t*-test. (F, G) Apoptosis-associated metabolite peaks (MM14+Lip14/Cre [F] and MM09+Lip09/Cre [G]) in the hippocampus did not change during treatment (*P *> 0.05; one-way repeated-measure ANOVA). Data are presented as mean ± 1 SD.

Subjects between 18 and 40 years of age and newly diagnosed ALL confirmed by reference morphology and immunophenotyping to be treated according to the German multicenter study group for adult ALL (GMALL) protocol (GMALL 07/2003; ClinicalTrials.gov: NCT00198991) (Registry[Bibr b16]) were enrolled at the Department of Hematology and Oncology between March 2010 and January 2012. Exclusion criteria comprised previous chemotherapy, neuropsychiatric diseases, CNS-acting medication or other relevant conditions interfering with the study protocol. GMALL07/2003 protocol includes a 7 weeks treatment period with preventive intrathecal administration of Methotrexate and Dexamethasone, oral treatment with 6-Mercapopurin and parenteral administration of Cyclophosphamide, Vincristine, Daunorubicine and Cytarabine as well as 24 Gy whole brain radiation (Fig.1A) (Registry). The study was approved by the local institutional review board (EK153052009) and all participants gave written informed consent.

Ten of 12 screened patients met eligibility criteria, gave informed consent, and went through the protocol as outlined in Figure1A. Of the total number of patients, 4 (40%) were female and 6 (60%) were male, mean age ± SD [range] was 28.04 ± 6.07 [19–36] years. All patients suffered from newly diagnosed ALL without brain pathology as determined by cerebral MRI and clinical examination and were allocated to the GMALL 07/2003 protocol including preventive brain radio-chemotherapy (Registry).

Subjects were examined on a 3.0T MRI system (GE Health Care, Munich, Germany) at three time-points: before (1), during (2) and 3 weeks after (3) ending of radio-chemotherapy (Fig.1A). Three orthogonal sets of T2-weighted images were acquired for positioning of volumes of interest (VOI). Single-voxel spectroscopy was performed in right hippocampus as well as parieto-occipital cortex as a reference (nonneurogenic) region. A point-resolved spectroscopy (PRESS) sequence (TE = 35 ms, TR = 1500 ms, NEX = 384) was used. Relative concentrations of total creatine, choline-containing substances, lactate and lipids at 1.3 and 0.9 ppm were estimated using LCModel software 3.0 (Provencher 1993). To minimize systematic errors between single MRS measurements we used creatine (Cre) as an internal reference as described before (Sartorius et al. 2003). We did not detect significant differences in Cre concentrations in the two regions during radio-chemotherapy indicating that Cre is a reliable reference metabolite.

We employed the computerized VWM as used previously to analyze spatial memory in humans (Bartsch et al. 2010; Schoenfeld et al. 2014). Subjects performed the test before (T1), during (T2, T3) and after the CNS radio-chemotherapy (T4) as outlined in Figure1A. The test comprises of a virtual island, on which a treasure box has to be located. Subjects virtually navigate on the island from a first-person perspective. Four orthogonal cues were located offshore. The experiment itself consisted of the following parts: (1) A training trial allowing the participants to get used to the joystick and computer display. (2) Six acquisition trials per day for 4 days (T1–T4; Fig.1A), in which participants had to find the hidden target. The start position varied across learning trials. (3) At time points T1 and T3, a recall test was performed 30 min after the learning trials with removed target. Outcome measure was the time spent in the original target quadrant. At time points T2 and T4, after 30 min delay, six reversal learning trials with the target hidden in a new position. We chose this alternating design as it has been suggested that adult hippocampal neurogenesis is particularly important for flexibility in spatial learning (Garthe et al. 2009) and to minimize a learning effect which is solely attributed to repetition of the initial acquisition trials. At the beginning of each trial, the patients always received the same instruction to find the hidden treasure but no further information about modifications of the task irrespective of whether the position has been changed or the target has been removed. (4) Three control trials with a now visible target served as an assessment of visuo-motor control (VMS). Quantitative measures included for each trial were heading error measured in degrees and total distance moved measured in proportion to total pool diameter.

Within-subject comparisons of ^1^H-MRS and VWM data were calculated using two-sided paired *t*-test or one-way repeated measure ANOVA with post-hoc Bonferroni-adjusted two-sided *t*-test as appropriate. Data were analyzed using the software programs SPSS 21.0 (SPSS Inc., Chicago, IL). All data are displayed as means ± SD or numbers (%), significance level was set at *P* < 0.05 (two-sided test).

## Results

### Brain radiation and chemotherapy lead to hippocampus-specific changes of the choline-signal in MR-spectroscopy

The presents of proliferating stem cells and migrating precursor cells in neurogenic regions of the adult brain may change the metabolite profile measured with MR-spectroscopy. We particularly focused on the choline peak, as changes of choline signal are suggested to be associated with altered cell membrane mobility that could be observed in proliferating cells and other plasticity associated events like neurite outgrowth and synaptogenesis (Araki and Wurtman 1997). MR-spectroscopy was performed in the right hippocampus and parieto-occipital cortex as a reference region that does not contain adult stem and precursor cells and therefore is claimed as a nonneurogenic region. Creatine (Cre) was used as an internal reference metabolite (for more details see Subjects and Methods section).

We found a higher tCho/Cre ratio in the hippocampus compared to the cortex of untreated patients (Fig.1B–D, *P* < 0.001), indicating a higher degree of membrane turnover in this region. Interestingly, the tCho/Cre ratio declines during brain radio-chemotherapy within the hippocampus, but not in the cortex (Fig.1E). After a 3-week interval without any cytostatic treatment, the tCho/Cre ratio in the hippocampus partially recovered (Fig.1E). We did not observe alterations in other metabolite signals, particularly there were no changes in metabolites associated with cell death (lactate and lipids at 0.9 and 1.3 ppm; Fig.1F and G) indicating no alterations in cell membrane turnover due to cell death.

### Brain radiation and CNS-chemotherapy does not affect performance in a spatial memory task (virtual water maze)

A functional relevance of newborn neurons could have been demonstrated for some but not all aspects of spatial memory formation. At least for rodents there is evidence that the addition of new neurons in the adult hippocampus is important for the flexible integration of new information into existing contexts (Garthe et al. 2009). To prove this hypothesis for humans we tested different aspects of spatial memory performance in a “humanized” version of the reference memory task of the Morris water maze.

**Figure 2:**
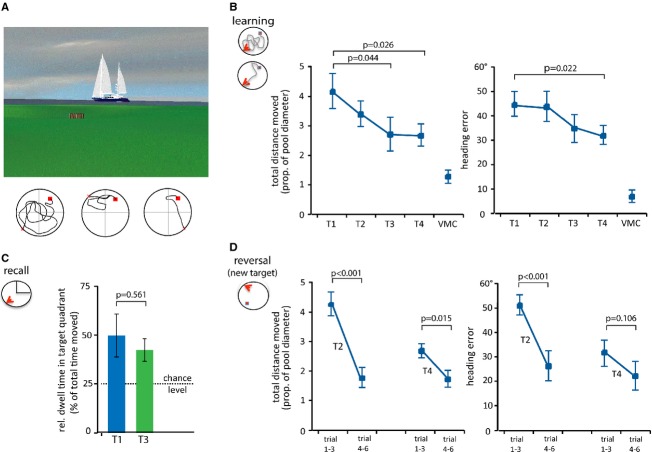
Virtual Morris Water Maze performance in brain radio-chemotherapy. (A) View of the island with the treasure hidden in a hollow and a sailing boat as one of the four external cues. Lower panel shows the path of trial 1–3 of one patient. (B) Diagrams show performance data of learning test displayed as total distance moved measured in proportion to the total pool diameter (left panel) and as heading error in degrees (right panel) as mean values of six trials for each day (T1–T4). Visuo-motor-control (VMC) reflects performance with a visible target. One-way repeated measure ANOVA of the intent-to-treat population revealed significant differences between time points (heading error: *F*-value = 3.219; *P *= 0.038; total distance moved: *F*-value = 8.098; *P* = 0.001). (C) At time point T1 (before) and T3 (after radio-chemotherapy), a recall test has been performed 30 min after the learning trials. Here the target (treasure) was removed from the virtual island. Bars indicate relative dwell time in target quadrant. The dashed line at 25% represents the random chance level. Both values (at T1 and T3 time point) are different from chance level with *P* < 0.05 (two-sided one-sample *t*-test). (D) Reversal learning was tested by changing the position of the target at time point T2 and T4. Learning performance is displayed as distance moved (left panel) and mean heading errors (right panel) for trial 1–3 and trial 4–6. Data are presented as mean ± 1 SD. *P*-values in diagrams are from Bonferroni adjusted post-hoc two-sided paired *t*-test (B) or two-sided paired *t*-test (C, D).

We found no evident abnormalities in the gradually improved learning performance during the longitudinal investigation at four time points before, during and after brain radio-chemotherapy (T1–T4). Heading error and total distance moved significantly declined during the learning trials at the four time points (Fig.2A and B). To identify a possible impact of the treatment on either the recall of the learned position or reversal learning of a new target position in the same environment, we included two alternating tasks with either a removed target (recall task at T1 and T3) or a new target position (reversal learning at T2 and T4). We did not observe any differences in the recall performance measured as relative dwell time in the target quadrant at the end of the radio-chemotherapy period (T3) compared to baseline (T1, Fig.2C). Flexible (reversal) relearning of a new target position did not show any impairment after the treatment period, but rather a better performance in finding the target in the first trial at the later time-point (Fig.2D). Together, performance in the spatial memory task as measured by repeated VWM test did not change during or after brain radio-chemotherapy.

## Discussion

The present longitudinal study using ^1^H-MRS and Virtual Morris Water Maze testing in patients undergoing preventive whole brain radio-chemotherapy demonstrated that CNS radio-chemotherapy provoke hippocampus-specific decrease in choline as a metabolite associated with cellular plasticity (proliferation) in ^1^H-MRS, but did not alter specific aspects of spatial memory function as measured by the VWM.

In the past, ^1^H-MRS had raised the hope of some scientists to visualize or detect adult neural stem cells in the human brain (Manganas et al. 2007), but the assumed stem cell specific peak at 1.28 ppm turned out not to be actually specific for a cell type but the cell state or more precisely apoptosis (Loewenbruck et al. 2011; Park et al. 2014). However, we think that the metabolite profile detected with MR-spectroscopy might still be a powerful tool to trace cell proliferation and cellular plasticity in specific brain regions. tCho as measured by ^1^H-MRS is composed of glycerol phsosphocholine (GPC), phosphocholine (PC) and to a negligible small amount of free choline (Barker et al. 1994). The main metabolites GPC and PC are involved in cell membrane synthesis and degradation making tCho in ^1^H-MRS a well-established method to detect highly proliferative brain-tumors. Indeed, a strong correlation between the tCho signal and the number of cells expressing the proliferation marker Ki67 was demonstrated (Steele and Morris 1999). Together, our findings suggest that tCho detection with ^1^H-MRS is sensitive enough to measure distinct alterations in cellular plasticity (neurogenesis) within the adult human hippocampus. This view is supported by a previous study showing a decreased tCho signal within the hippocampus of patients with major depression with presumably reduced hippocampal neurogenesis, which is elevated by electroconvulsive therapy (Ende et al. 2000), a procedure reported to be a robust stimulator of hippocampal precursor cell proliferation in mice (Jessberger et al. 2005). However, the specificity of the tCho signal for neurogenesis is not entirely clear. ^1^H-MRS signals could typically not be cell type- specific markers as reliably distinguishable signals generally compound of metabolites that indicate changes of the cell status. GPC and PC become detectable whenever cell membranes are synthesized or depleted reflecting mainly cell proliferation or cell death and to some extent synaptogenesis, neurite outgrowth (Araki and Wurtman 1997) and possibly migration. Specificity of ^1^H-MRS signals could be enhanced by analyzing the composition of various other peaks, in our case other cell death-associated signals such as lactate, and various lipid-peaks also reflecting membrane compounds known to be elevated in case of cell death (Loewenbruck et al. 2011). However, we found no changes in lactate or lipid-peaks (at 1.3 and 0.9 ppm) indicating no changes of cell death in the observed brain regions by the used radio-chemotherapy protocol. Together, our ^1^H-MRS results point to a hippocampus-specific, radio-chemotherapy-induced reduction in cell membrane synthesis most likely reflecting neurogenesis. The described changes in ^1^H-MRS-based metabolite profiles might be a novel approach to monitor adult neurogenesis in humans.

During recent years, it became more and more clear that adding new neurons to the hippocampal network facilitates important aspects of memory formation (e.g. pattern separation), but this process seems not to be indispensable for other basal aspects of memory formation. It has thus been postulated that the specific design and interpretation of the memory test to be used is of great importance. In our study, we adapted the original rodent learning task (Morris water maze) that has been successfully used to detect neurogenesis-specific aspects (Garthe et al. 2009) to a human longitudinal study design. This modification also includes aspects of the delay-matching-to-place task (DMP), which is often used in longitudinal study designs before and after an intervention (Steele and Morris 1999). Although the reliability of the used VWM to test hippocampal function in humans has been proven before (Bartsch et al. 2010; Schoenfeld et al. 2014), it is unclear whether the adaption to a longitudinal study design would still have the power to detect neurogenesis-associated changes in hippocampal function. The improvement during learning trials from T1 through T4 is naturally due to the repetition of the task. Due to the lack of a control group we could only assert that patients were generally able to learn the target position but could not evaluate the quality of learning performance. However, in animal studies blocking neurogenesis leads to specific changes in spatial-memory performance affecting particularly the encoding of new but similar memory into preexisting networks. We therefore included a six trials-test 30 min after the initial learning trials where the target position has been changed at two time points during the study: at the beginning of the therapy (T2) and 3 weeks after the end of the therapy (T4). This reversal-test alternated with the recall-test where the treasure box has been removed from the island at T1 and T3. As the patients did not know that the target position only change at T2 and T4 and was removed at T1 and T3 it can be assumed that performance in the reversal-learning task and recall trial cannot be influenced by repetition. The patients in our study not only show a similar performance at trials 4–6 of the reversal task at the two time points, but also an even better first trial learning after changing the position at T4. This last observation is comparable to the DMP task where first trial learning of a changed target position has been found to be a hippocampus-dependent learning paradigm (Steele and Morris 1999). We therefore suggest that brain radio-chemotherapy did not affect the flexible integration of new memory into a preexisting context at least during the time frame of 3 weeks after the radio-chemotherapy. In rodents, it takes about 3–4 weeks after the initial cell division until the newborn neuron is functional integrated into the hippocampal network (Toni et al. 2008), in humans this relevant information is still lacking. Thus, it is possible that even the latest time point in our approach was still too early to test functional aspects of inhibited neurogenesis. Future studies including a variety of hippocampal learning tasks tested in a larger cohort at different time points are required to more precisely answer the question of the functional relevance of adult human neurogenesis.
